# Has Athletic Performance Reached its Peak?

**DOI:** 10.1007/s40279-015-0347-2

**Published:** 2015-06-21

**Authors:** Geoffroy Berthelot, Adrien Sedeaud, Adrien Marck, Juliana Antero-Jacquemin, Julien Schipman, Guillaume Saulière, Andy Marc, François-Denis Desgorces, Jean-François Toussaint

**Affiliations:** IRMES (Institut de Recherche bioMédicale et d’Epidémiologie du Sport), INSEP, 11 avenue du Tremblay, 75012 Paris, France; EA 7329, Université Paris-Descartes, Sorbonne Paris Cité, Paris, France; Frontiers in Life Science E.D. 474, Paris, France; CIMS, Hôtel-Dieu, Assistance Publique des Hôpitaux de Paris, Paris, France

## Abstract

Limits to athletic performance have long been a topic of myth and debate. However, sport performance appears to have reached a state of stagnation in recent years, suggesting that the physical capabilities of humans and other athletic species, such as greyhounds and thoroughbreds, cannot progress indefinitely. Although the ultimate capabilities may be predictable, the exact path for the absolute maximal performance values remains difficult to assess and relies on technical innovations, sport regulation, and other parameters that depend on current societal and economic conditions. The aim of this literature review was to assess the possible plateau of top physical capabilities in various events and detail the historical backgrounds and sociocultural, anthropometrical, and physiological factors influencing the progress and regression of athletic performance. Time series of performances in Olympic disciplines, such as track and field and swimming events, from 1896 to 2012 reveal a major decrease in performance development. Such a saturation effect is simultaneous in greyhound, thoroughbred, and frog performances. The genetic condition, exhaustion of phenotypic pools, economic context, and the depletion of optimal morphological traits contribute to the observed limitation of physical capabilities. Present conditions prevailing, we approach absolute physical limits and endure a continued period of world record scarcity. Optional scenarios for further improvements will mostly depend on sport technology and modification competition rules.

**Electronic supplementary material:**

The online version of this article (doi:10.1007/s40279-015-0347-2) contains supplementary material, which is available to authorized users.

## Key Points

**Table:** 

Sport performance in athletic species has reached a peak in numerous disciplines.
Performance is limited by physiological and genetic conditions, economic and environmental contexts and a participant population consisting mostly of athletes with optimized traits.

## Introduction

Whether and when sport performance reaches its natural limit is a subject of considerable debate and disagreement among experts [1]. An article in 2004 [2] gave rise to a lively debate in the academic field. It stated that linear models could be used to predict the progression of human performance in sprint races in the twenty-second century. As arguments favoring and opposing such a methodology were discussed, subsequent publications empirically argued that the progression of sport performance instead follows a non-linear trend [3–6]. These projections are based on data (e.g., speed, distance, height) gathered in various competitions from the late nineteenth to the early twentieth century. These enormous amounts of data contain best performances and records of elite athletes and provide a historical perspective on the development and progression of athletic performance since the re-introduction of the modern Olympic Games. After more than 100 years of active athletic competition, the processes leading to peak performance have been carefully optimized by athletes, trainers, and supporting staff who turned to professionalism. However, athletic capacity is also influenced by biology [7–10] and environment [11–14] in addition to physical, biochemical, and ecological rules, plus a touch of chance. The goal of this review was to detail the working hypothesis that human capabilities cannot progress indefinitely and have reached a state of stagnation. Progression among other animal species is also documented in order to provide support for the hypothesis. Finally, this work suggests directions to overcome the observed limits in sport performance.

## Limitation of Performance Progression

**Fig. 1:**
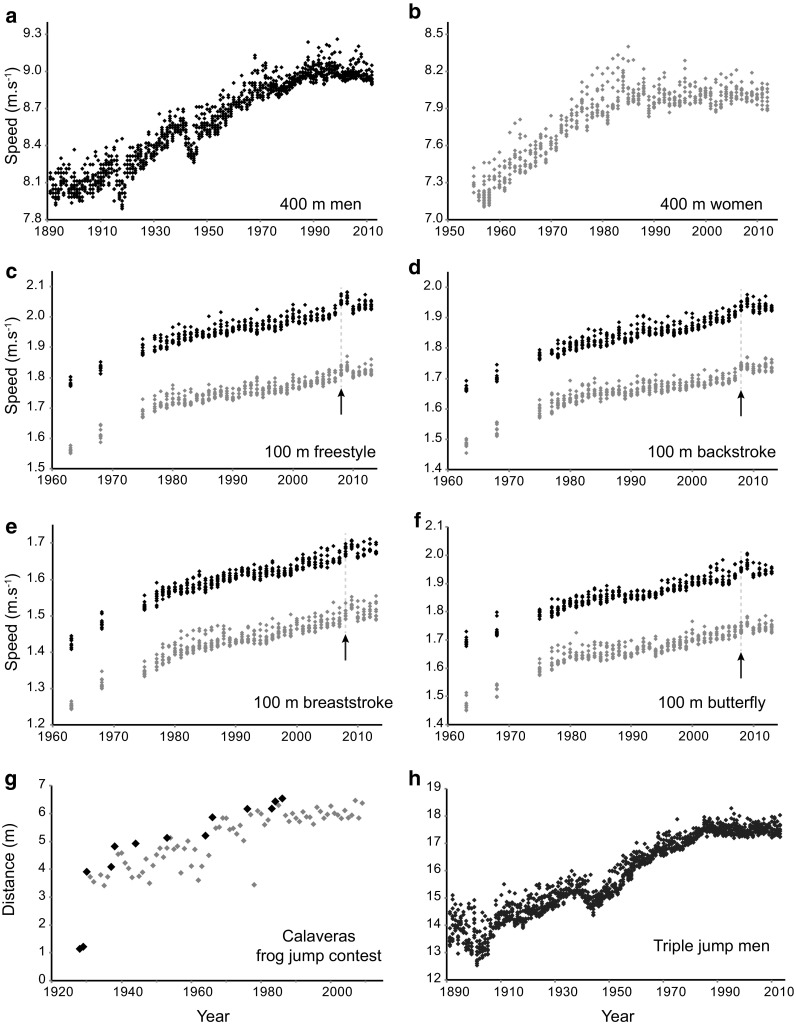
Best performances over time in **a** men’s 400 m; **b** women’s 400 m; **c** men’s and women’s 100 m freestyle swim; **d** men’s and women’s 100 m backstroke swim; **e** men’s and women’s 100 m breaststroke swim; **f** men’s and women’s 100 m butterfly swim; **g** records (*black diamonds*) and annual best performance (*grey diamonds*) in the Calaveras frog jump contest; and **h** men’s triple jump. Values shown are the single best result of the yearly top ten world performers for human competition (with *black diamonds* representing values for men and *grey diamonds* representing values for women). The *black arrows* indicate the introduction of polyurethane swimsuits in 2008, and the Olympic cycle is observable (with a ≈1 % progress every 4 years [17])

Since the introduction of the modern Olympic Games in 1896, the development of sport performance has been investigated in many aspects of the scientific literature. Various mathematical models have been introduced to describe and predict future performance. One study in particular triggered controversy among experts [1, 2]: a linear model was adjusted to the development of world records (WRs) in track and field 100 m straight. The author suggested that the WR would follow a constant progression rate, and eventually lead to an instantaneous 100 m in a distant future, with infinite speed. Other authors chose a more physiological approach and used piecewise exponential or non-linear sigmoid models [3–6, 15]. They provided more reasonable estimates of both actual progression and future performance. Exponential and sigmoid models were not designed to describe performance development prior to the 1900s. They produce abnormal performance values: negative or constant marks (i.e., times or distances recorded in competition) and thus do not explain previous evolution. Here we focus on the recent plateau of performances and observe that non-linear models are (1) accurate in describing recent performance development and (2) converge toward a common paradigm: future performance will not progress indefinitely. To determine the progression of athletic performance as comprehensively as possible, we investigated all 147 quantifiable Olympic events during the modern Olympic era [15]. Five disciplines were included in the study: track and field, cycling, speed skating, weight lifting, and swimming. A total of 3263 WRs were gathered, starting in 1896. A decrease in both the frequency and the relative improvement of WRs was discovered, revealing a major decrease in progression, suggesting an exponential—thus limited—development with time (Fig. 1). The pattern also unveiled a step-wise progression related to various technological, pharmacological, or sociological improvements. In addition to Olympic events, a similar result was observed in the analysis of WR development in outdoor events exposed to non-standardized environments [16]. Ten famous non-Olympics events, including boat, speed skating, or country ski races were investigated. Some of these events—such as the Oxford–Cambridge rowing race—have kept competition records since 1829, providing exceptional data on historical follow-up and trends. All the competition results recorded were largely influenced by the environmental conditions and material or technical constraints. The study revealed that the shape of progression was identical in standardized and non-standardized events, despite large differences in effort duration, environment (hot, cold, hypoxic, windy), or media (ice, snow, water, ground, air) [16].

Although WR is a good representation for demonstrating the progression of ultimate human capacities, it provides limited insights into the performance of athletes who are not record holders. To investigate a more accurate measure of physiology and its fluctuations over the past 120 years, we investigated the annual performance of the ten best performers (Fig. 1) [17]. More than 40,000 performances from 36 track and field and 34 swimming events were characterized. Again, performance progression was associated with a decrease in recent years, in line with the WR results [3–6, 15]. However, the 100 m men and marathon may still have a limited margin of progression. Exceptional athletes like David Rudisha, Dennis Kimetto, and Geoffrey Mutai have recently established new WRs, but they do not alter the observed paradigm for the yearly top ten marks (Fig. 1 and Electronic Supplementary Material [ESM] Appendix S1).

As described above, human sport performance appears to have plateaued in recent times. But do the performances of other species also display a plateau? Is there a common rule for progression? Such a hypothesis was challenged as a similar analysis was performed using data gathered in official greyhound and thoroughbred competitions [18]. Dogs and horses are two examples of species selected for their athletic capacities and trained during past centuries. Other species such as frogs can be seen competing in jump contests (Fig. 1). For all species, overall performance progression was similar to that observed in human events, with a sigmoid pattern [18]. A similar pattern displaying a stagnation in performance progression appeared for various physiological functions (jumping, swimming, running) in classes as different as batrachians (Fig. 1g) and mammals. This process may be the common rule of performance development in species that are placed in a competitive environment. Using data collected from over a century and simple mathematical models, various authors have shown that all studied species are now approaching their performance limit [3–6, 15–18].

## Determinants of Performance Stagnation

Progression of performance is an irregular process (Fig. 1); it is altered by natural and artificial conditions. Athlete preparation conditioning, including physical training, nutrition, and medicine, was affected by technological improvements and innovations in the twentieth century. Fogel [19] coined the term “techno-physiological evolution” to describe anthropometric gains over the last 3 centuries. However, the most noticeable parameters influencing performance relate to biology (including genetics [7, 8, 20–23]: *ACTN3 R577X* [7, 24], *HFE* [the gene that encodes the HFE protein or human hemochromatosis protein], genes coding for myostatin, adrenergic-*β*2 or erythropoietin [EPO] receptors [8]; and time-dependent processes such as growth and aging [9, 10, 25–27]); and environment [11–14], which can be either natural (e.g. ambient temperature, gas content, barometric pressure, winds) or human based (through cultural [28] and technological [29] contexts). Not all of the factors involved have been identified yet, but some conditions could explain the recent scarcity of new WRs.

### Technological Innovations

In the last decades, relatively large performance growths in swimming, cycling, speed skating, speed skiing, and sailing were largely dependent on technological improvements [29]. Innovations can lead to a rather large progression step: the highest performance recorded by the International Human Powered Vehicle Association in the hour speed cycling event was associated with a speed of 91.6 km h^−1^ using streamlined recumbent bicycles. In comparison, the official record of the Union Cycliste International (UCI), despite new rules allowing lenticular wheels, is still almost twice as low at 51.1 km h^−1^ [16]. In road cycling, the development of records is related to the introduction of specific technologies, such as duralumin, carbon fibers, and aerodynamic handlebars [30]. Another measurable impact of technological advances is the swimsuits introduced in 1999 [31]. The effect of the three successive generations of suits on elite swimmers over the 1990–2009 period was measured. The use of these high-tech products led to a 3 % mean progression before their 2010 ban [31] (Fig. 1c–f).

Pharmacological innovations produced performance-enhancing drugs based on products initially developed to compensate for defective physiological functions in patient populations. EPOs, growth hormones, steroid hormones, or amphetamines have made history in many sporting disciplines [32]. To better understand the historical relationship between these drugs and performance progression, we used a statistical approach to measure and score outlying ‘atypical’ values in the track and field and swimming datasets of top performers. Atypical progressions were observed in 1943 (all sports), 1988 (all sports), 1993 (track and field), and 1994 (swimming) [17]. In the 1993 post-Olympic year, Chinese female athletes achieved exceptional performances and were responsible for 33 % of the top performances, 33 % of the second performances, and 39 % of the third performances. These ratios have never been equaled by China or any other country since. That same year, at the National Games in Beijing, five Chinese women beat the 3000 m running WR, a unique moment in the whole of sport history. This record has never been approached since. In swimming, the year 1994 corresponded to another doping affair: seven Chinese athletes were declared positive at the 1994 Asian Games in Japan. Yesalis and Bahrke [33] questioned the role of East German coaches in China’s sport programs after the fall of the Berlin Wall. The use of doping substances may explain the last burst of performance occurring in the 1990s [17, 32–35]. Talented athletes may have set insuperable marks through the use of doping drugs. Such levels of performance may produce demotivation for subsequent competitors, for which solutions may exist [36]. Stronger anti-doping policies, institutions, and out-of-competition screening tests have been in place since then and may also have a place in the explanation of the present stagnation. New statistical approaches such as the biological passport may further help in limiting future occurrences of ultra-physiological performances [37–39]. However, these methods are scarce and debated [34, 40–42]. Finally, the ability to maintain funding for such doping control activities will no doubt influence future performance levels.

Every effective innovation results in a rapid improvement in athletic performance, and each technological ban has been followed by a downward shift of the best athletic capacities [16, 31] (Fig. 1c–f). Without technology, the level of many disciplines would not have risen to the current standard. However, money, through the cost of technology, may act to raise the next limitation step. In fact, the cost of the polyurethane swimsuit ($US400) was one of the major arguments in the 2009 FINA (Fédération Internationale de Natation or International Swimming Federation) decision to ban these innovations.

### Morphology

**Fig. 2:**
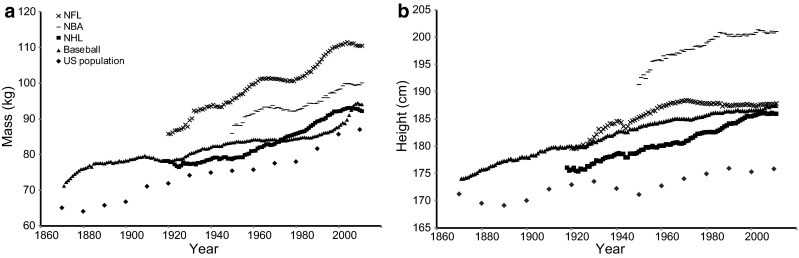
Evolution of mean **a** mass (kg) and **b** height (cm) of National Basketball Association (NBA), National Hockey League (NHL), National Football League (NFL) and baseball players [43]

Morphological parameters, including body mass and height, increased in the populations during the twentieth century [19], leading to taller, heavier, and stronger athletes in most sporting disciplines [43, 44]. Morphology is an important factor for determining the proper selection of sporting events [43, 44]. The morphological enhancement of athletes followed the same piecewise exponential pattern observed in numerous sports records [15] (Fig. 2). For example, morphological increases in sprinters closely mirrored their speed improvement [44]. The level-off of morphological growth in athletes, probably due to the exhaustion of a selective process in the largest pool of athletes, may be another reason the progression of WRs has stagnated in recent years [43]. In basketball and track and field events, a reduction in variability in anthropometric traits can be observed as the performance level increases [43, 44], and the best athletes now gather around optimal ranges of height or body mass index (BMI). A number of other anthropometric traits have been identified over the past few decades. For example, the reciprocal Ponderal Index (a measure of linearity) has emerged as an important indicator of success in elite sprinters [45] and appears to be a useful indicator in the categorization of footballers in successful teams [46]. Anthropometry illustrates the best trade-off between different requirements in a multi-objective optimization problem, revealing complexity in determining athletic performance.

### Phenotypic Selection

Athletes with more adapted phenotypes have gradually taken their place among the best performers during past decades [47]. African men and women runners are increasingly dominating the marathon races and extending its records. Recent doping affairs and networks do not totally explain such a progression trend over more than 20 years. Producing intense aerobic or anaerobic efforts, mastering complex techniques or strategies, today’s best performers and record holders have phenotypes that closely fit the specific constraints of their discipline. Such precise adaptations are more clearly observable in American football (National Football League [NFL]), basketball (National Basketball Association [NBA]) [43], and track and field [44]. One obvious limit may then rise from the exhaustion of new demographic pools, when all potential phenotypes have been detected, selected, and trained.

### Sociocultural Influence

**Fig. 3:**
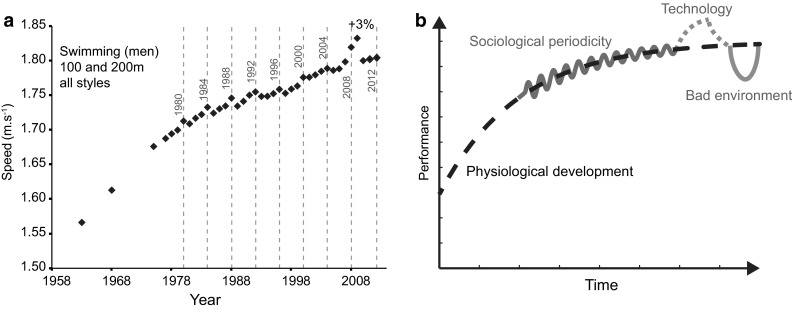
Mean performance data for men in **a** swimming. Each *black diamond* represents the mean value of the top performance of the 80 best performers (100 and 200 m freestyle, butterfly, breaststroke, and backstroke in swimming, and 100 m to marathon in running). In **a**, Olympic periodicity is shown in the swimming events (*grey lines* and *dates*). A gain of around 3 % was established after the introduction of polyurethane swimsuits. **a** is summarized in a conceptual **b** depicting the physiological (*black dashed line*), sociological (*wavy grey line*), technological (*grey dashed line*), and environmental (*grey solid U*-*shaped line*) cycles of development

The human-made environment impacts progression of sport performance. Major socio-cultural events such as the Olympic Games have a significant periodic impact (around 1 % every 4 years, Fig. 3) on the top ten performers [17]. The analysis of the performance of the top 50 athletes in the 100, 200, 400, 800, and 1500 m running races also showed that one of the two yearly peaks in performance was indeed related to the calendar of international competitions (Olympic Games, World and European Championships) [14]. The periodicity of these cycles is not influenced by a global trend in physiological progression nor by its recent slowdown (Figs. 1c, d, f, 3). However, the amplitude of the peaks may shrink as performances approach their limit (Fig. 3). Additional social effects may take place as media and the audience (both on TV or internet programs) have progressively become a leading factor for sport promotion. Less popular events may therefore experience athlete and sponsor defection. When looking at the historical progression of WR, one can observe that both world conflicts sustainably altered performance progression (Fig. 1) by 6.4 and 13.4 years, respectively [15], whereas the Cold War accelerated the progression rate on both East and West sides [28]. Thus, it is expected that future major historical events may possibly alter both the organization of international competitions and the developments of physical performance.

### Environment and Periodicity

**Fig. 4:**
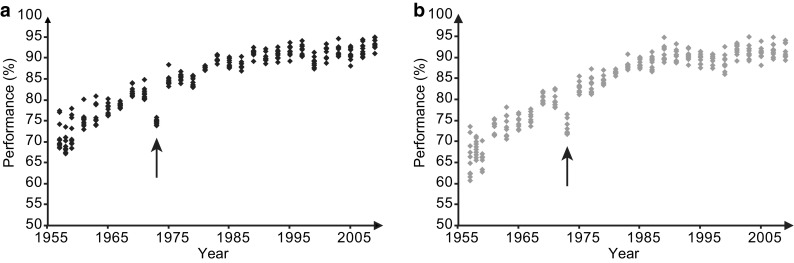
Ten best performances at the outdoor world archery championship for **a** men and **b** women between 1957 and 2009. Due to the change in the number of arrows allowed in competition, we normalized the performances as the ratio of the observed score against the maximum possible score each year. The decrease in top performances in archery in 1973 (*black arrows*) was due to very poor environmental conditions with heavy local storms

Competitions performed outdoors follow a similar progression toward stagnation [16] despite a larger dependence on ambient temperature, barometric pressure, oxygen concentration, wind (Fig. 4) or humidity. Ambient temperature has been identified as a major parameter limiting global physical performance, in both short-distance [14] and long-distance races [13, 48]. Warm weather is known for its detrimental effect on distance runners as it limits thermoregulatory control [11, 48], but cold conditions also tend to reduce athletic performance [12]. The relationship between climate and performance was extensively analyzed in the marathon: a comprehensive survey of the six largest marathons worldwide (Paris, London, Berlin, Boston, Chicago, and New York) from 2001 to 2010 was performed by El Helou et al. [13]. The relationship between 2 million chronometric results values and environmental factors (including humidity, dew point, pollutants) showed that air temperature was the most significant parameter, in both male and female runners, whatever their performance level. The optimal temperature to run a marathon is about 10 °C [13], and for sprint and middle distances it is around 23 °C [14]. Various scenarios from international institutions (United Nations [UN], Intergovernmental Panel on Climate Change [IPCC]) indicate a higher average temperature in areas where major sporting events are to be held in the future. These scenarios may possibly further alter their organization and might delay performance progression. Other environmental effects, such as wind velocity and turbulence, are associated with detrimental impacts on progression of rowing [16] or archery performance (Fig. 4), highlighted that degraded environmental conditions may negatively affect further performance development.

### Economic Factors

Economic conditions will play a major role in upcoming years as it has both local and global impact on the organization of sport institutions. For example, the annual frequency of WRs was impacted by the Great Depression in the 1930s [15]. A durably degraded economic context coupled with the growth of aging populations may shift economic resources away from sports organizations [49]. Countries and national federations may then restrict financing support to a limited number of disciplines and athletes in order to secure the only medals that count in the nations’ competition. International sport would then be structured in specialized niches, with the majority of countries only competing in selected disciplines. This would defeat the original spirit of the Olympic Games that promoted sport as a universal and popular feature. Economic development also interacts with population growth and the global performance of societies in both directions, such that one may include a degraded economic, environmental, and climatic context in upcoming scenarios with eroded societal infrastructures in order to sketch a perfect storm.

### Random Effects

Randomness may affect sport performance at all possible levels, from inter-individual genetic variation to intra-individual cyclic variabilities. Performance of elite and non-elite athletes may exhibit fluctuations related to random events [17]. Injuries, illnesses, and familial or social tensions may impact the individual athletic career, while technological innovations, and geopolitical and environmental events usually affect the performances of the whole population (Fig. 1). In contrast, ‘chance’ may have a positive acute impact on personal records. An example of such an atypicality is Bob Beamon’s 1968 long jump. It suddenly altered the development of WRs in this event, producing an outstanding value, unbeaten for 22 years. When compared with the rest of his career or with the jumps of all the other Mexico finalists during their entire career, such a performance never occurred again [17].

## Can we Break Performance Stagnation?

The elements previously detailed provide the key points for a scenario of interactions determining the progression of sport performance, where technology and environment and societal influences add to the principal phenotypic and physiological trend (Fig. 3). Despite all these enhancements, performances start to plateau in a vast majority of events (see Fig. 1 and the additional data provided in ESM Appendix S1). However, a number of ways exist in which performance stagnation can be overcome. For example, new resources (such as nanomaterials), artificial tissues, or designs may help produce new athletic records. One may also choose to refine chronometry and distance or height measurements. Times recorded in milliseconds, and jumps in millimeters, will artificially produce new WRs, but only resample the observed stagnation. New regulations can also produce new records. Sport institutions can possibly allow for the use of abandoned or banned technologies, such as sliding rigger boats in rowing. They can also alter specific rules such as the underwater distance that produce greater swimming speeds, or the mass and profile of thrown objects (e.g., javelin). Creation of new disciplines will produce new WRs as illustrated by the inclusion of female events in weight lifting in 1998. This strongly increased the annual frequency of new WRs in subsequent years [15].

But none of these options will change the rules of performance progression nor the physiological regulations. However, altering human genetics (today’s hopes and hype [23]) may result in artificially modified performances, even if major ethical considerations will most likely prevent such manipulations (since 2003, the World Anti-Doping Agency [WADA] and the International Olympic Committee [IOC] have placed gene doping among prohibited methods). However, the lack of success in gene therapy, despite 30 years of research and considerable effort [50], may delay applications in sport for the upcoming years.

## Conclusions

Performance in humans and animals (frogs, greyhounds, and thoroughbreds) has experienced a plateau in the last 20–30 years. Physiological, environmental, historical, societal, and economic aspects are among the parameters that may contribute to such stationary behavior. However, technological innovations may alleviate the observed stagnation, depending on the evolution of rules and regulations.

## Electronic supplementary material

Supplementary material 1 (PDF 2532 kb) **Online Resource 1** Figure compiling the top-ten performance development with time in 37 track and field events (including the 1 mile men) for men and women
