# Supplementary material for: Has Athletic Performance Reached its Peak?
Source: Sports Med. 2015 Jun 21;45(9):1263–71. doi: 10.1007/s40279-015-0347-2 (PMC4536275; doi:10.1007/s40279-015-0347-2)

Has athletic performance reached its peak ?

Sports Medicine

Geoffroy Berthelot[1,2], Adrien Sedeaud[1,2], Adrien Marck[1,2,3], Juliana da Silva Antero-Jacquemin[1,2], François Denis Desgorces[1,2], Guillaume Saulière[1,2], Andy Marc[1,2], Julien Schipman[1,2], Jean-François Toussaint[1,2,4]
1 - IRMES (Institut de Recherche bioMédicale et d'Epidémiologie du Sport), INSEP, Paris, France
2 - EA 7329, Université Paris-Descartes, Sorbonne Paris Cité, France
3 - Frontiers in Life Science E.D. 474, 8-10, rue Charles V, 75004 Paris, France
4 - CIMS, Hôtel-Dieu, Assistance Publique - Hôpitaux de Paris, Paris, France
Contact Information:
G Berthelot, geoffroy.berthelot@insep.fr, 11 avenue du Tremblay 75012 Paris

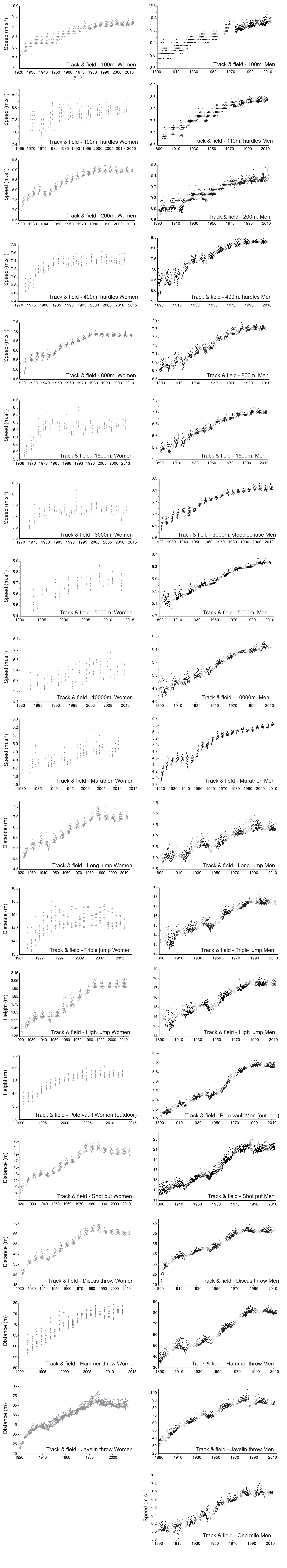

Supplement: Supplementary file 1 — Supplementary material 1 (PDF 2532 kb) Online Resource 1 Figure compiling the top-ten performance development with time in 37 track and field events (including the 1 mile men) for men and women [file 40279_2015_347_MOESM1_ESM.pdf]
